# Electron probe microanalysis as a versatile tool to probe the chemistry of radioactive wasteforms

**DOI:** 10.1557/s43577-026-01195-8

**Published:** 2026-10-01

**Authors:** Stuart Creasey-Gray, Martin Stennett, Clare Thorpe, Garry Manifold, Brant Walkley, Charlotte Nevin, Raúl Fernández, Jonathan Morgan, Robert Harrison, Lewis Blackburn

**Affiliations:** 1 School of Chemical, Materials and Biological Engineering, University of Sheffield, Sheffield, S1 3JD UK; 2 Department of Geology and Geochemistry, Faculty of Sciences, Autonomous University of Madrid, Cantoblanco, 28049 Madrid, Spain; 3 Department of Mechanical and Aerospace Engineering, Nuclear Fuel Centre of Excellence, Sir Henry Royce Institute, The University of Manchester, Manchester, M13 9PL UK

**Keywords:** Immobilization, Wasteforms, Plutonium, EPMA, Microscopy, Characterization

## Abstract

Electron probe microanalysis (EPMA) is a powerful, nondestructive microscopy tool that finds ubiquitous application in the broader field of materials science. Herein, it was demonstrated that EPMA is a versatile tool for probing the chemistry of nuclear materials, particularly wasteform systems for the immobilization of intermediate and high-level radioactive waste, with constituents such as Cs, Sr, U and Pu. This communication evidences the efficacy of EPMA to further understand the five distinct wasteform systems for both nonradioactive surrogate material particularly relevant to the UK radioactive waste management and disposal program, including alpha-active systems containing ^238^U.

**Graphical abstract:**

**Figure:**
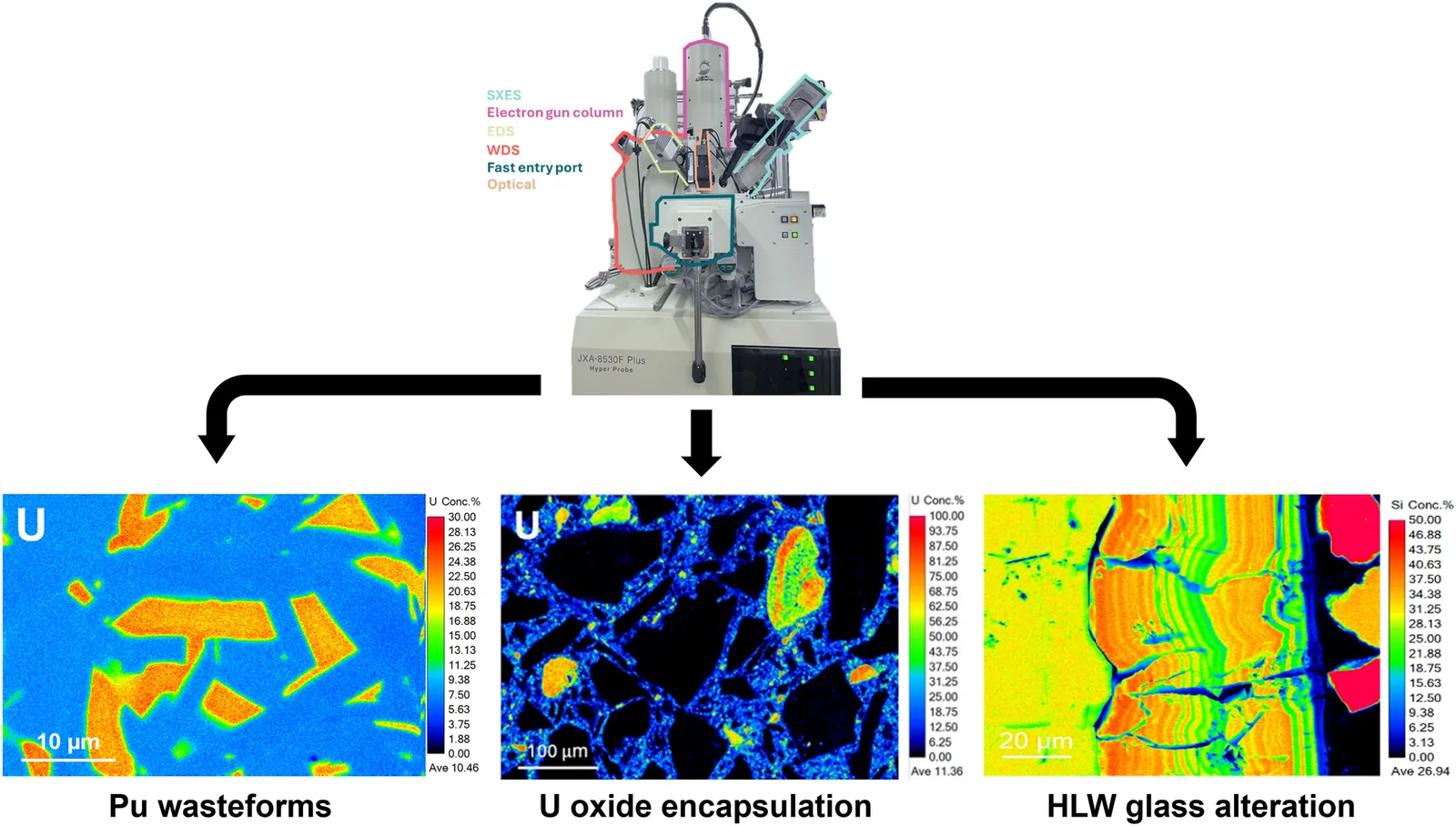

## Background

The safe, responsible and effective management of radioactive waste, generated as a byproduct from either civilian or defence related activities, presents one of the 21st centuries most substantial technical and societal challenges and has been the subject of many international research programs. For contaminated wastes containing long-lived isotopes, isotopes with a particularly high specific activity, or otherwise elevated radiological, biological or chemical hazard, immobilization processes can improve the handling and storage properties, and facilitate disposal. Broadly, immobilization processes may be thermal (i.e., vitrification), chemical (ceramic solid solution), or physical (encapsulation) yet each process has the ultimate goal of creating a passively safe ‘wasteform’ package that chemically and/or physically retards the migration of harmful radioisotopes over geological time scales within the broader generic geological disposal concept.^1–4^

The UK has a long and complex legacy of waste production, with the most recent 2025 UK Radioactive Waste Inventory estimating a packaged inventory of 1340 m^3^ high-level waste (HLW) and 618,000 m^3^ intermediate-level waste that will be consigned to a geological disposal facility (GDF).^5^ In the UK, the waste inventory for disposal can broadly be categorized into high heat generating wastes (HHGWs) typically comprising spent nuclear fuels (e.g., pressurized water reactor [PWR], advanced gas-cooled reactor [AGR], Magnox, Windscale advanced gas-cooled reactor [WAGR], boiling water reactor [BWR]), vitrified high level wastes (e.g., MW Magnox, Ca/Zn blend, post operation clean out [POCO]), immobilized plutonium products (undefined product specification yet proposed as either disposal mixed oxide [DMOX] or a hot isostatically pressed titanate ceramic) or low heat generating wastes (e.g., irradiated graphite, ferrous metals, sludges/flocs, zircaloy, ion-exchange resins, depleted uranium tails). While some materials (e.g., spent fuel) will not be chemically reprocessed and will be overpacked for disposal, many waste streams (e.g., calcined high level wastes, separated plutonium, POCO) will require immobilization prior to disposal. Designing an immobilization flowsheet and producing materials that fall within an expected product performance envelope requires extensive R&D to design appropriate host matrices, and many microscopic, spectroscopic or diffraction-based techniques are used to evaluate material chemistry and performance.

## Wasteform design

The concept of a ‘wasteform’ is material with elevated radioactivity (either through transmutation/activation, contamination) that is physically, chemically, thermally, or otherwise treated to alleviate the difficulties associated with interim or long-term storage, transport, and disposal. Ultimately, the objective is to bind radioactive species within the host material either through encapsulation (i.e., providing a physical barrier) or chemical immobilization (i.e., atomic-scale substitution of radionuclides within a host material). The philosophy underpinning the design of a wasteform strategy or process is to tailor the chemistry of the host matrix to the properties of the waste itself, while optimizing (among other properties): wasteloading, chemical durability, radiation stability, and mechanical robustness.^6–8^ Each wasteform system and accompanying immobilization technology has its relative advantages and disadvantages with considerations to temperature constraints, simplicity, scalability, compatibility with waste acceptance criteria for disposal, and so on; for example, cementitious grouts are relatively facile to produce, do not require high temperatures and are cost-effective at an industrial scale, hence is a suitable system for encapsulating low heat generating wastes that do not have intrinsically high thermal or radiation fields. Whereas, a high-performance ceramic system such as pyrochlore (Gd_2_Ti_2_O_7_) has exceptional tolerance to radiation, high chemical durability yet requires high temperatures and is not routinely produced at scale. Several authoritative reviews on wasteform design and selection have been published in recent years and the authors refer the reader to the following prominent works.^2,3,9–11^

## Electron probe microanalysis

Electron probe microanalysis (EPMA) provides a significantly enhanced means to obtain precise and accurate quantitative elemental compositional analysis of complex materials systems at the micron scale.^12^ EPMA complements the visual inspection of the microstructure performed in an SEM by providing quantitative information about the chemical elements present under the electron beam. It answers key questions for many applications in materials science: what elements are present in microstructural features, how are they spatially distributed, and what are their concentrations.

When considering applications to radioactive materials analysis, as with conventional EPMA, careful sample preparation is required to obtain reliable quantitative data. Samples should ideally present a flat, homogeneous and well-polished surface, typically prepared by sectioning, mounting, grinding, and sequential polishing to a sub-micron finish. Nonconductive materials are generally carbon coated to minimize charging during analysis. These requirements can present additional challenges for radioactive materials, where sample mass, size, contamination control, remote handling, containment, and radiological dose constraints may restrict conventional metallographic preparation. Consequently, preparation procedures must be compatible with the relevant radiological controls, and the amount of material removed during sectioning and polishing should be minimized while still producing a representative analytical surface. The examples presented herein use preparation routes appropriate to the individual wasteform systems.

**Figure 1:**
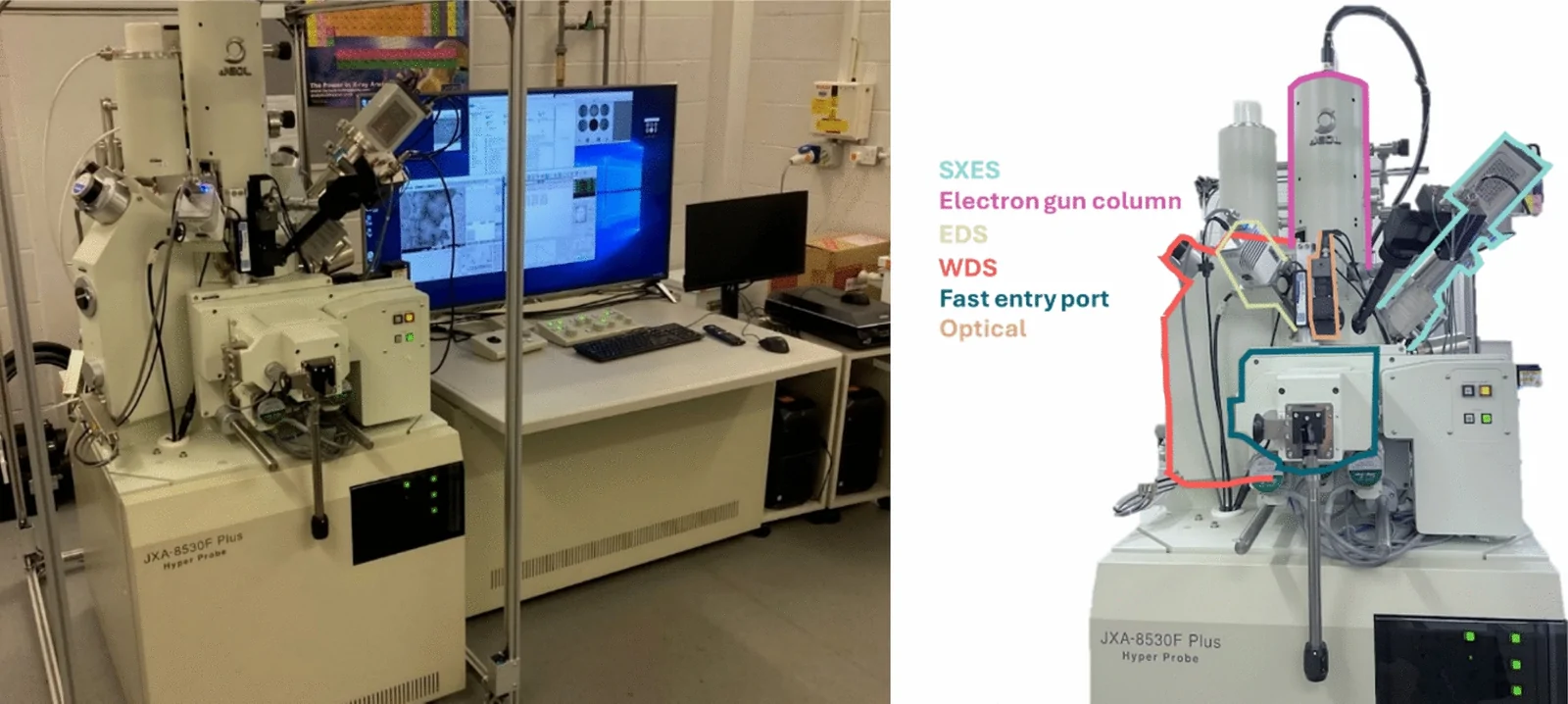
JEOL JXA 8530F Plus electron probe microanalyser (EPMA) hosted at the Sorby Centre for Electron Microscopy at the University of Sheffield.

**Figure 2:**
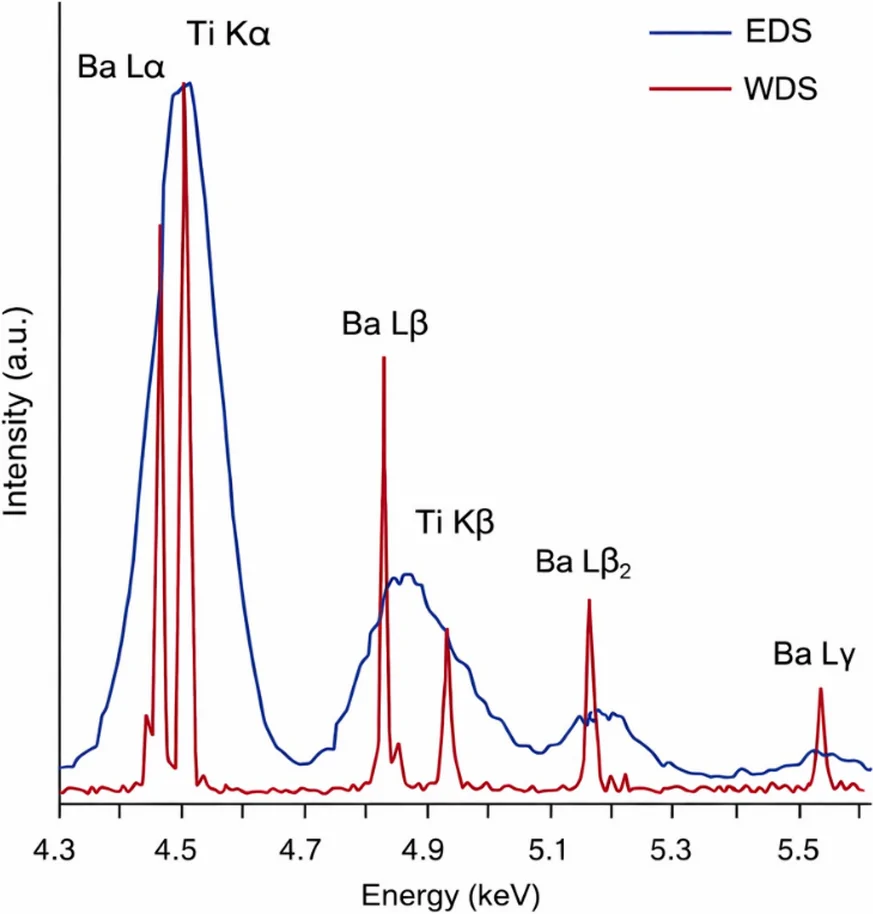
Enhanced spectral resolution for wavelength dispersive spectroscopy (WDS) compared with energy dispersive spectroscopy (EDS) for Ba and Ti.

Supported by the Henry Royce Centre for Advanced Materials and using materials largely produced within the High Activity Decommissioning Engineering and Science (HADES) National Nuclear User Facility^13^ at the University of Sheffield, UK, analysis of various wasteform systems has been achieved using a JEOL JXA 8530F Plus consisting of a FEG gun system, which provides higher resolution imaging than conventional tungsten filament electron microscopes (**Figure** **1**). It is also equipped with four crystal spectrometers for wavelength dispersive spectroscopy (WDS) and a detector for energy-dispersive spectroscopy (EDS). This configuration enables a greater spectral resolution by WDS enhancing the analytical capabilities that can be achieved using conventional EDS alone (**Figure** **2**), with sensitivity down to ppm levels. In EDS, the characteristic X-rays generated by the electron beam are detected and separated according to their photon energies. WDS instead uses an analysing crystal to diffract X-rays according to their wavelength, following Bragg’s Law, with the crystal and detector geometry selecting a narrow wavelength interval for measurement. The improved spectral resolution is particularly valuable where characteristic X-ray lines overlap, allowing individual elements to be distinguished and improving peak-to-background ratios, detection limits and quantitative accuracy.

This configuration affords a bespoke analytical setup for almost any element combination (B to U) in solid matrices relevant to wasteform systems, including: minerals, glasses, cements, metals and alloys, and ceramics. This analytical capability, combined with data processing software, form a very powerful and cost-effective tool for academic and industrial research. It is recognized, however, that the capital and operational costs of a dedicated EPMA are substantially greater than those of a conventional SEM equipped with EDS. Consequently, EPMA is not necessarily the most appropriate technique for all radioactive materials applications (particularly for transuranic materials where appropriate reference standards may be difficult to acquire) and SEM–EDS can therefore present a more accessible and cost-effective solution where rapid analysis and compositional screening is required. The principal advantages of EPMA arise where high precision quantitative analysis, trace element determination or resolution of closely spaced X-ray emission lines is required. Instrument calibration is performed by analysing reference materials of known compositions. The choice between SEM–EDS and EPMA should therefore be determined by the required detection limit and precision, sample activity and available infrastructure. A major part of the analysis is to determine for each element of interest, which X-ray line with their order of reflection to use to minimise the effects of peak interference and overlaps on analysis. The accurate evaluation of peak scans (profiles) and determination of background positions provide accurate and reproducible results, with routine detection limits typically 60–600 ppm, depending on the element and host material.

Electron microscopy techniques such as compositional back scattered electron imaging can be used to identify regions of interest, which then can be quantitatively analyzed, and elemental compositional maps and/or line scans can be obtained. Hence, EPMA provides a powerful analytical tool, which together with other techniques such as SXES and electron backscattered diffraction (EBSD) allows detailed materials characterization to be undertaken at the microstructural level. The JXA 8530F Plus is also equipped with a soft X-ray emission spectrometer (SXES) enabling ultrahigh spectral resolution, even better than WDS, offering the potential for chemical state mapping particularly of light elements. This is particularly of interest when exploring the influence of light elements such as boron in alloys and lithium or sodium in novel battery materials.

**Figure 3:**
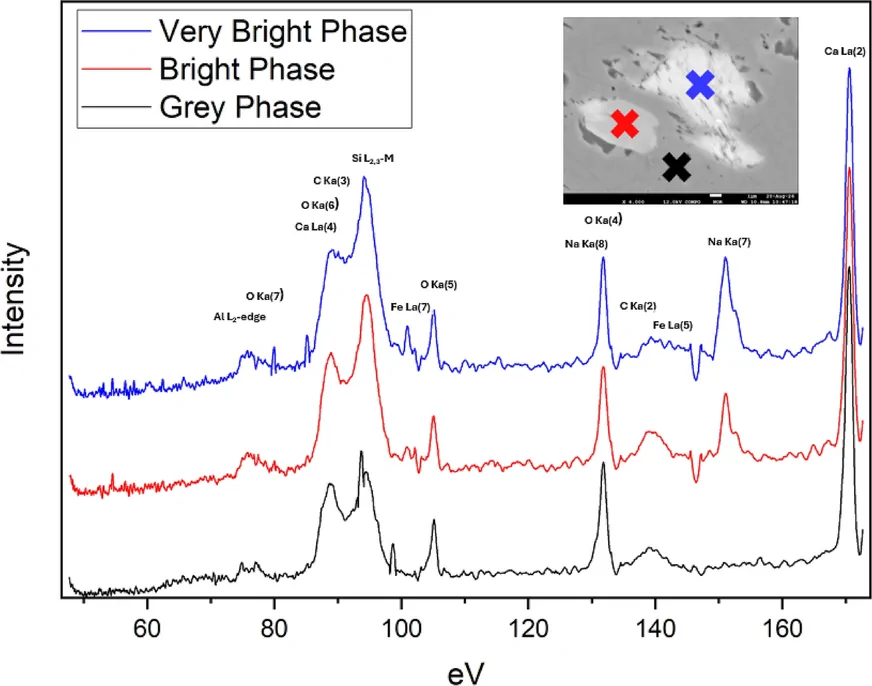
Soft X-ray emission spectroscopy (SXES) of various phases contained within a specimen of jadeite.

The SXES spectrometer uses X-ray focusing optics, variable line-spacing gratings (JS50N and JS200N) and a solid-state X-ray CCD detector to collect a low energy X-ray spectrum. It provides higher spectral resolution and higher sensitivity for soft X-rays (50–210 eV) relative to traditional WDS, yielding information on electronic structure and materials bonding from examining the peak shapes. The grating and CCD detector are rigidly fixed, with no moving parts, resulting in spectra that are extremely reproducible. This allows for the analysis of ultralight elements with very high peak-to-background ratios. An example of SXES spectral scans of different phases within Jadeite (NaAlSi_2_O_6_) are shown in **Figure** **3**.

EPMA offers high spatial resolution for quantitative analysis and is therefore powerful tool in the arsenal for radioactive materials analysis as it only necessitates a small interaction volume within the material. The electron beam interaction volume in EPMA varies depending on the accelerating voltage used, the material composition and density, and the porosity of the material being analyzed. Typical interaction volumes, defined here as containing approximately 80% of the simulated electron trajectories using CASINO software, were calculated for UO_2_ as approximately 0.15 µm at 10 kV, 0.18 µm at 12 kV and 0.23 µm at 15 kV. These interaction volumes also influence the depth from which characteristic X-rays can escape, although the effective escape depth is strongly dependent on the energy of the X-ray and the composition of the surrounding matrix. Low-energy (soft) X-rays, such as those from light elements, including C, N, and O, are more readily absorbed by heavier matrices and therefore have substantially shallower effective escape depths than higher-energy (hard) X-rays. The spectral resolution of WDS is 8 eV, which is significantly greater than that for EDS (120–130 eV), which provides better peak overlap resolution. The JEOL 8350F EPMA is equipped with a Schottky source field emission gun (FEG), resulting in a highly focused (40–100 nm) electron beam that allows both imaging and analysis at sub-micron level to be undertaken at lower kV. EPMA is thus capable of high spatial resolution and relatively high analytical sensitivity, which compliments and surpasses other surface analytical techniques such as EDS analysis and hence, determine element concentrations in spots as small as 0.1 µm diameter down to concentrations 30–40 ppm.

The interaction of the electrons with the sample produces characteristic element specific X-rays typically K_α_, L_α_, M_α_, M_β_… which are diffracted to produce wavelengths intensities on a known set of analyzing crystals as part of a WDS. The WDS support various analyzing crystals such as LIF/LIFL, PET/PETL, TAP/TAPL, and layered dispersion elements (LDE1, LDE2, LDEB) for light element optimization. The wavelength intensities are counted by an X-ray detector to produce an electric signal. This signal is proportional to the number of X-rays, which is also proportional to the concentration of the element “i” in the sample. The electrical signal is then amplified and compared to the signal produced in the same conditions by a known standard. The final concentration of the element “i” in the sample is calculated based on the proportionality between the X-ray intensity in the unknown sample and the X-ray intensity produced by the known standard, following a correction procedure to minimise the “matrix” effect on the measurement. A range of known standards are selected to perform elemental calibrations with selection of peaks and background positions matching those for the analysis of the sample. Great care is needed to ensure that the peaks and backgrounds do not interfere with other elements and if so, intensity correction factors are obtained. The standards used ideally must be of similar known composition to that of any unknown or use pure elements. The standards and the unknown are run under the same beam conditions with appropriate matrix correction software applied such as ZAF and PRF. The peak intensities for each element of interest are run using pulse height analysis (PHA) to set the optimum gain, Hv (V), base level (V) and window (V). The following MAC reference standards were used with serial numbers for active materials 16,860 for Thorianite (Th, U)O_2_, ThO_2_ (5.9 = 5.18 wt%Th in glass); U_3_O_8_ (5.54 = 3.85 wt% U in glass). Other MAC reference standards for rare earths (serial no. 14019), minerals (serial no. 16778), geological (serial no.16777) and universal 45 (serial no. 14021) were used to select appropriate calibration material for each element and to check the accuracy of analytical data. Elements are directly detected and measured but not chemical bond state, there is however, an option to convert the elemental data into that for oxides by knowing their valence number. For example, from the measurement of the concentration (wt%) of element “i” in an unknown silicate, knowing the valence of the element “i”, the result can be calculated and reported as ”oxides.”

As working with radioactive materials presents intrinsic handling and size restraints, this presents an advantage for higher activity materials as it may reduce exposure risk, simplify shielding requirements and make it feasible to analyse high activity materials that would otherwise be difficult to probe at the bulk scale. Moreover, the sample preparation requirements compared to techniques such as FIB-TEM are relatively facile. The instrument described in this work, a JEOL JXA 8530F Plus, represents the EPMA capability available at the University of Sheffield during the experiments reported herein and is therefore described to provide reproducibility and context for the case studies. This particular instrument is now discontinued, and the discussion should not be interpreted as being specific to a single commercial platform. Current EPMA developments include field-emission systems such as the JEOL JXA-iHP200F, which integrate WDS and EDS with automated analytical workflows and optional complementary techniques. The continued development of field-emission sources, detector technology, automated analysis and multimodal characterisation therefore provides a continuing role for EPMA in microchemical analysis of complex materials. The remainder of this article will highlight five short case studies wherein EPMA has been utilized in support of the development of nuclear wasteforms relevant to the UK’s current waste disposal effort.

## Immobilization of separated Pu in a zirconolite ceramic

As of 2025 the UK has adopted a strategy of immobilisation to manage its inventory of separated plutonium (c. 141 t) and now seeks to identify a preferred wasteform system and accompanying immobilization technology.^14^ Zirconolite (nominally CaZrTi_2_O_7_) has been the subject of much academic scrutiny and is a favorable choice based on its exceptional chemical durability, structural flexibility with regards to cation substitution and moderate radiation tolerance.^10,15–17^ Zirconolite manifests a number of polytype structures (2M, 4M, 3T, 3O) that are generally characterized by variation in stacking order of adjacent Ca/Zr and TiO_x_ layers in the base structure along the *c*-axis. The controlling factor in polytype stabilization appears to be a combination of (1) cation doping across Ca^2+^, Zr^4+^ and Ti^4+^ sites and (2) partial oxygen pressure during synthesis. At present, no product specification for a Pu-loaded zirconolite material is defined within the context of the UK Pu disposal effort, yet it is generally accepted that a bulk product containing ~20 wt% Pu comprising a single (or near single) zirconolite phase assemblage is preferable, and the optimisation of zirconolite solid solutions to satisfy these criteria (among others) is the subject of several ongoing research programs in the UK and elsewhere.^18^ The necessary engineering controls to work with even relatively small quantities of plutonium are highly constrained, hence when undertaking compositional scoping experiments that require a relatively high throughput and wide variation in stoichiometries, there is often a need to use chemical surrogates such as Ce, U, and/or Th.

Using UO_2_ as a surrogate for PuO_2_, a zirconolite ceramic with the nominal formulation CaZr_0.80_U_0.20_Ti_2_O_7_ was produced by planetary ball milling CaTiO_3_, ZrO_2_, TiO_2_ and UO_2_ oxides in masses appropriate to yield the desired stoichiometry. The mixture was agitated at 400 rpm for 20 min using isopropanol as a milling agent in a Y-stabilized ZrO_2_ lined milling vessel and 3-mm-diameter Y-stabilized ZrO_2_ media. The slurry was dried at 90°C and sieved to recover the powder, from which approximately 1 g pellets were produced by uniaxial pressing into a 10-mm hardened steel die and placed onto a ZrO_2_ crucible, which was then placed into a tube furnace. The material was sintered under a flowing argon atmosphere for a period of 20 h at a temperature of 1400°C, with programmed heating/cooling rates of 3°C/min. The pellet was polished to a 0.25 µm optical finish using various grades of SiC paper and diamond suspensions impregnated into polishing cloths.

**Figure 4:**
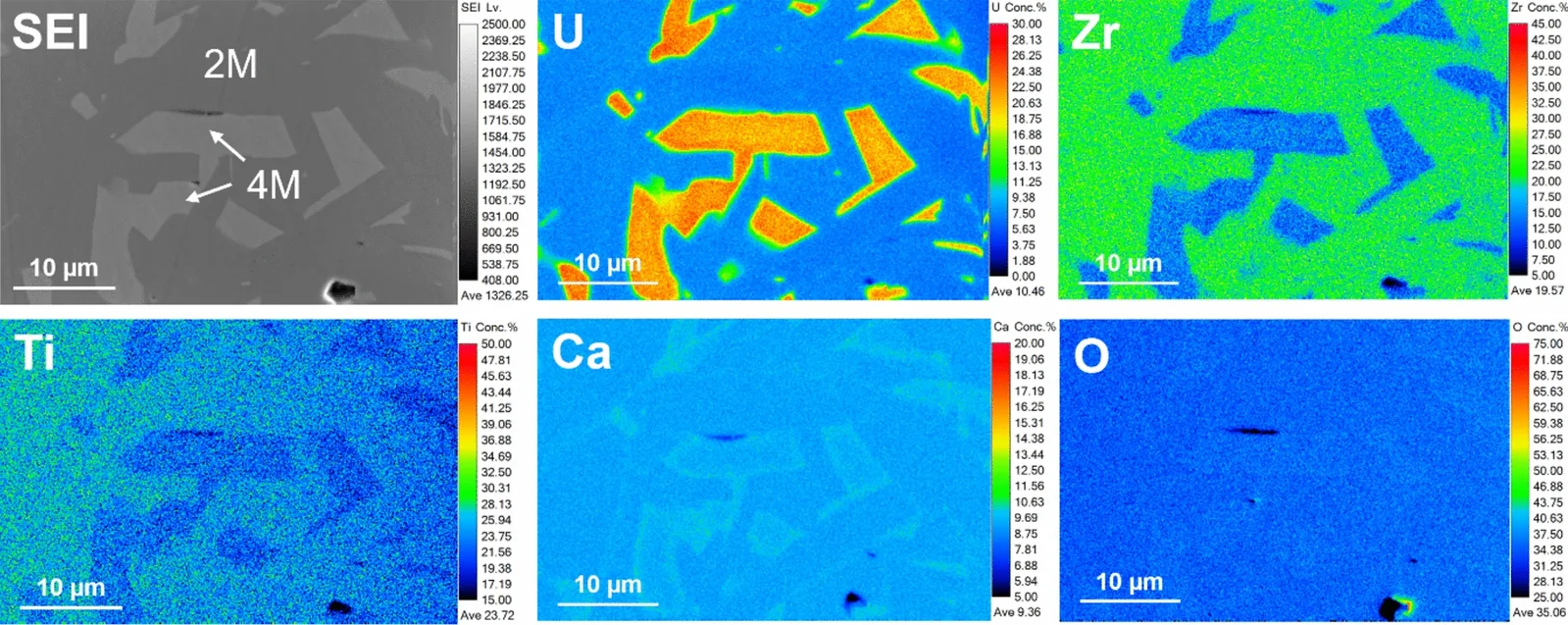
Secondary electron image (SEI) and corresponding electron probe microanalysis (EPMA) maps of CaZr_0.80_U_0.20_Ti_2_O_7_ zirconolite evidencing both 2M and 4M polytypes, with data presented as corresponding wt%.

The secondary electron images and corresponding composition maps for U, Zr, Ti, Ca, and O are presented in **Figure** **4**. Both the backscattered electron and secondary electron imaging function was used to locate appropriate area of the sample for analysis, yet the entire pellet surface comprised two compositionally distinct phases. The EPMA mapping function allowed the distribution of uranium, acting as a proxy to establish how plutonium would partition in the corresponding CaZr_0.80_Pu_0.20_Ti_2_O_7_ phase, to be mapped with high spatial accuracy. These data confirm that the U portion was preferentially distributed in grains of the zirconolite-4M polytype rather than the zirconolite-2M bulk phase. Quantitative EPMA demonstrated preferential U incorporation into the 4M phase, with nominal substitution levels of *x* = 0.124 and *x* = 0.362 for the 2M and 4M phases, respectively, based on the molar U/(U + Zr) ratio. Benchmarking the compositional limits at which zirconolite undergoes phase transformations is necessary in order to rationalise potential solid solution regimes for Pu and hence EPMA has the potential to be a powerful tool to probe the chemistry of more complex (Ca, Pu, Gd)(Zr, Pu, Gd, Hf)(Ti, M)O_7_-type systems where M is a charge balancing additive such as Fe or Al and Gd/Hf are present as neutron absorbers.

## DMOX surrogates manufactured using conventional and flash sintering

DMOX wasteforms present an attractive route for the disposition of separated UK plutonium.^19–23^ The concept builds on established fuel manufacturing process that blend UO_2_ and PuO_2_ prior to sintering; however, as there is no requirement to accommodate fission products or pore formers there is flexibility with regards to pellet geometry and there is no need for the pellets to meet the stringent physical dimension requirements for fuel fabrication. Current-rate controlled flash sintering (FS) is a novel sintering technique that offers significant time and energy savings over conventional sintering (CS) techniques^23–25^ However, assessing the viability of the technique for DMOX manufacture requires a broader understanding of how typical powder processing techniques influence the field and thermal effects that are widely responsible for the stabilised microstructures following sintering. EPMA was employed to map high-accuracy elemental concentrations of Ce at the micro-scale in (U, Ce)O_2–*x*_ DMOX surrogates, which could be paired with µ-Raman mapping to assess lattice strain related to oxygen defects present following sample manufacture.

Representative (U_0.93_,Ce_0.07_)O_2–*x*_ DMOX surrogate powder blends were fabricated following the principles of the short binderless route (SBR) and micronized master blend (MIMAS) techniques. DMOX pellets were sintered using CS (1700°C, 10 h hold, flowing Ar + 5 wt% H_2_) and FS techniques (ambient temperature of 600°C, 2 h, 101 mA/mm^2^ current density), then sectioned and polished to electron microscopy quality. Polished samples had fiducial markers ion milled into the surface before being coated with a 10-nm carbon coat using a Safematic CCU-010, prior to transfer to the Sorby Centre at the University of Sheffield with UO_2.00_ standards.

**Figure 5:**
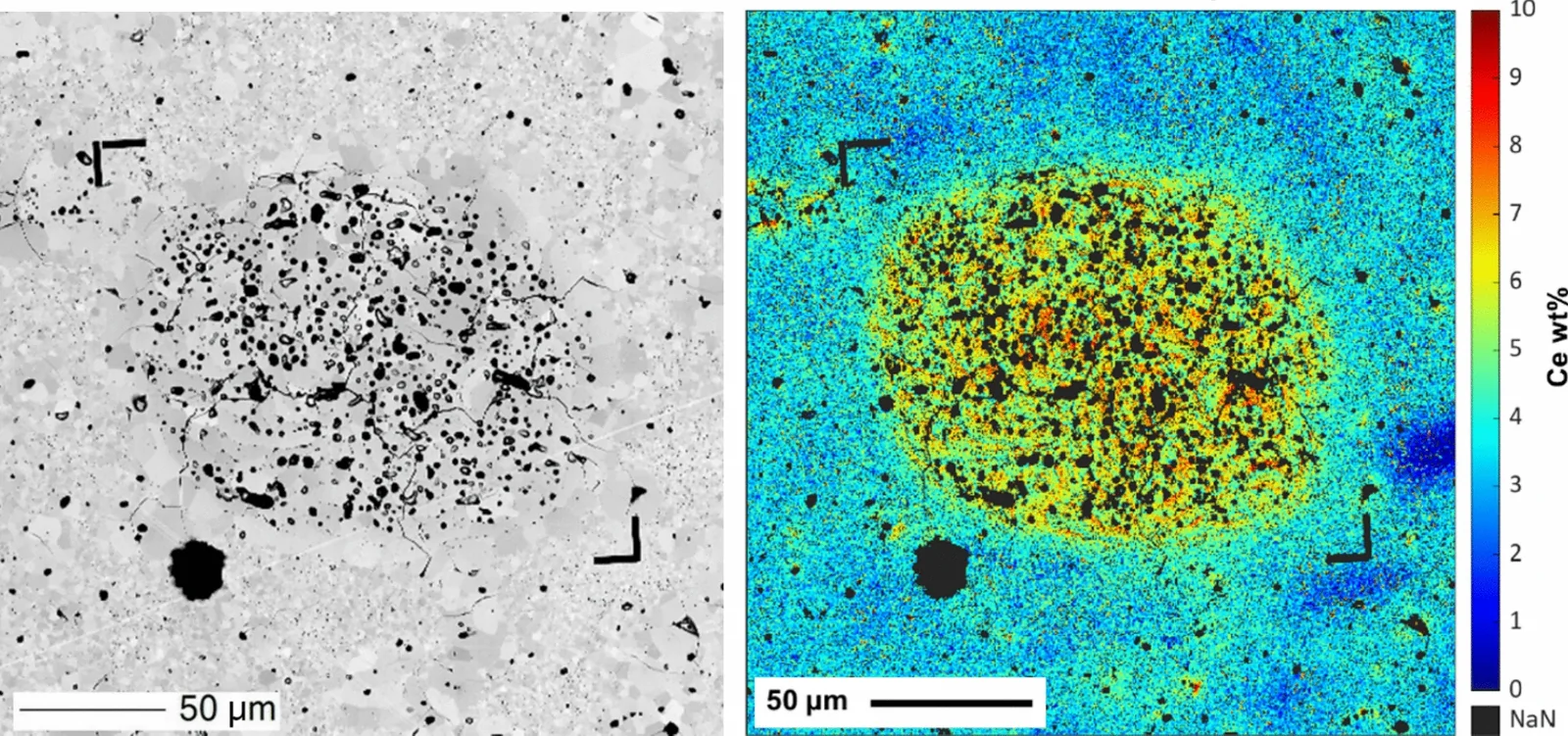
(Left) Backscattered electron (BSE) image of a residual island of high Ce loading master blend in a conventionally sintered Micronised Master Blend (MIMAS) pellet. (Right) the corresponding electron probe microanalysis (EPMA) map showing Ce wt% using the emitted intensity of the characteristic Ce L_α_ wavelength dispersive spectra (WDS) relative to a standard.

Representative elemental maps are shown in **Figure** **5**. Extended point scanning was additionally employed on features of interest to obtain high-resolution spectra for pseudo-quantitative localized determination of elemental composition. The resulting elemental determinations were used alongside µ-Raman maps collected from the same area, allowing pixel-by-pixel twinning of WDS and Raman spectra. This permitted direct comparison between the local elemental composition and lattice expansion. By knowing the local Ce content, the deviation in lattice parameter can be related to residual oxygen vacancies, allowing microscale studies of stoichiometry and oxygen potential in sintered DMOX surrogates.

## Development of geopolymer cement wasteforms for improved Cs and Sr retention

Geopolymer materials are proposed as promising alternatives to traditional Portland cement wasteforms for the immobilization of ^137^Cs and ^90^Sr. EPMA is particularly useful for investigating incorporation mechanisms of radionuclides in cement matrices and therefore was used to obtain high-resolution spatial distribution of elements and precise mass ratio quantification within the bulk matrix of geopolymer cements, before and after leaching experiments, to examine their suitability for encapsulation of radioactive waste bearing inactive isotopes of Cs and Sr,^26^ relevant to, for example, SIXEP at Sellafield, and the cleanup operation at Fukushima Daiichi Nuclear Power Plant. EPMA was specifically selected to circumvent limitations associated with oxygen interference on the elemental energy levels of caesium and strontium during standard energy-dispersive X-ray (EDX) analysis. Elemental maps and discrete point quantifications for Al, Si, Cs, Sr, Ti, K, and Na were acquired across multiple magnifications to characterise the geopolymer both prior to and following leaching. This enabled differentiation between phases bearing caesium and strontium and the chemical composition of the primary alkali aluminosilicate hydrate (K–A–S–H) gel (the main geopolymer binding phase) and localized features such as pores, unreacted metakaolin particles, and secondary precipitates.

**Figure 6:**
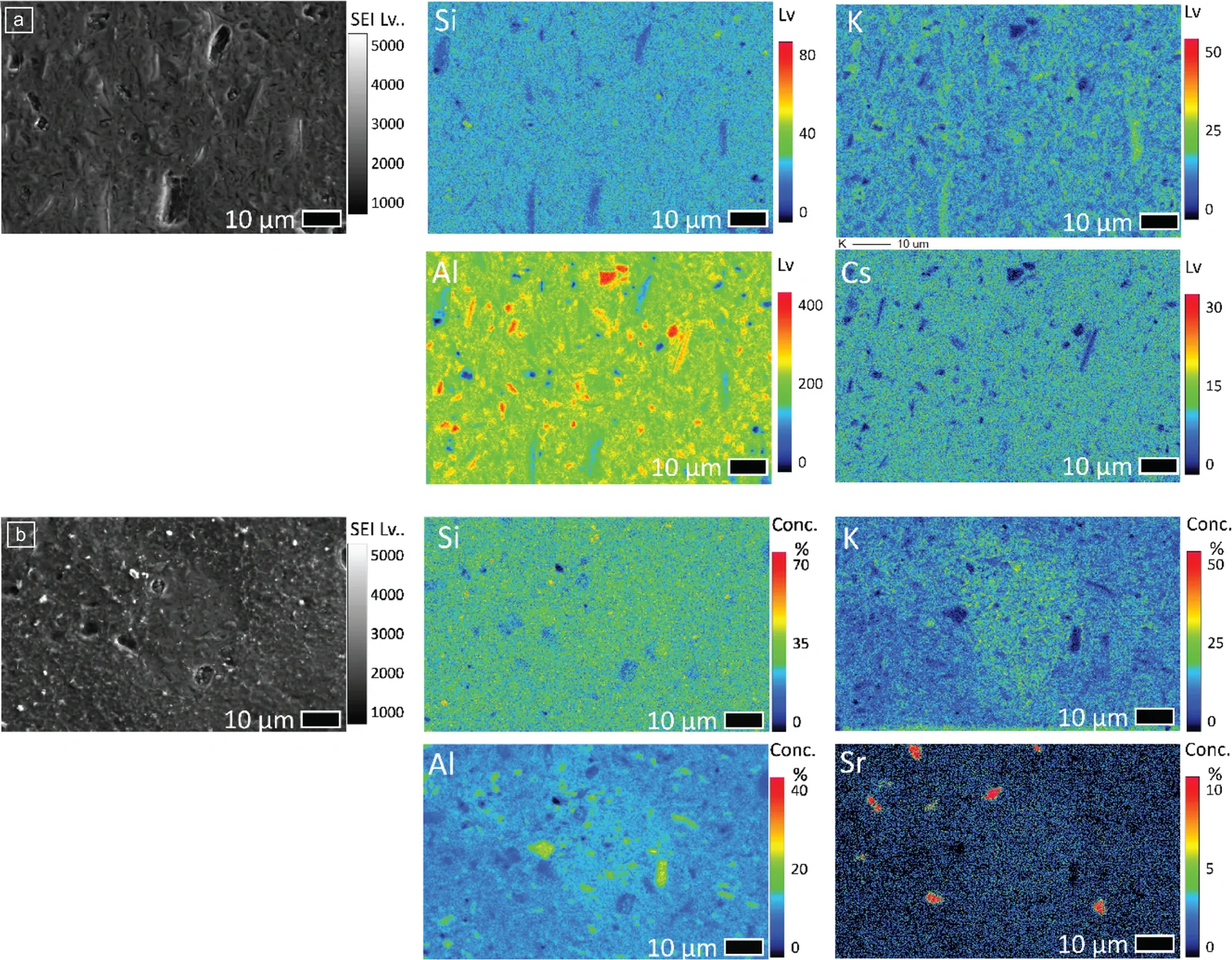
Electron probe microanalysis (EPMA) data for geopolymer cements with data presented as corresponding wt% containing (a) strontium (added to the alkali activating solution as 3 wt% strontium hydroxide octahydrate) and (b) caesium hydroxide (added to the alkali activating solution as 3 wt% caesium hydroxide).

The EPMA data revealed a generally homogeneous distribution of Si, Al, and K within the bulk geopolymer matrix, though significant heterogeneity was observed regarding the caesium and strontium distribution. In strontium-containing samples, Sr was present at approximately 1 mol% within the bulk geopolymer matrix, but also formed discrete regions with localized concentrations reaching up to 40 mol percent. These strontium-rich regions were depleted in Si, Al, and K, indicating the presence of undissolved Sr(OH)_2_ or the formation of SrCO_3_ precipitates. In contrast, caesium distribution was notably more uniform throughout the bulk geopolymer matrix, which is attributed to the higher solubility of CsOH under the reaction conditions. Whilst bulk caesium concentrations decreased significantly following the 35-day leach test, strontium levels remained relatively stable, corroborating the lower leachability of Sr observed in the aqueous phase analysis.^26^ Furthermore, EPMA identified titanium-rich clusters originating from the metakaolin precursor, which may have served as potential sorption sites for caesium (**Figure** **6**).

## Interaction of uranium oxides with MKPC phases

**Figure 7:**
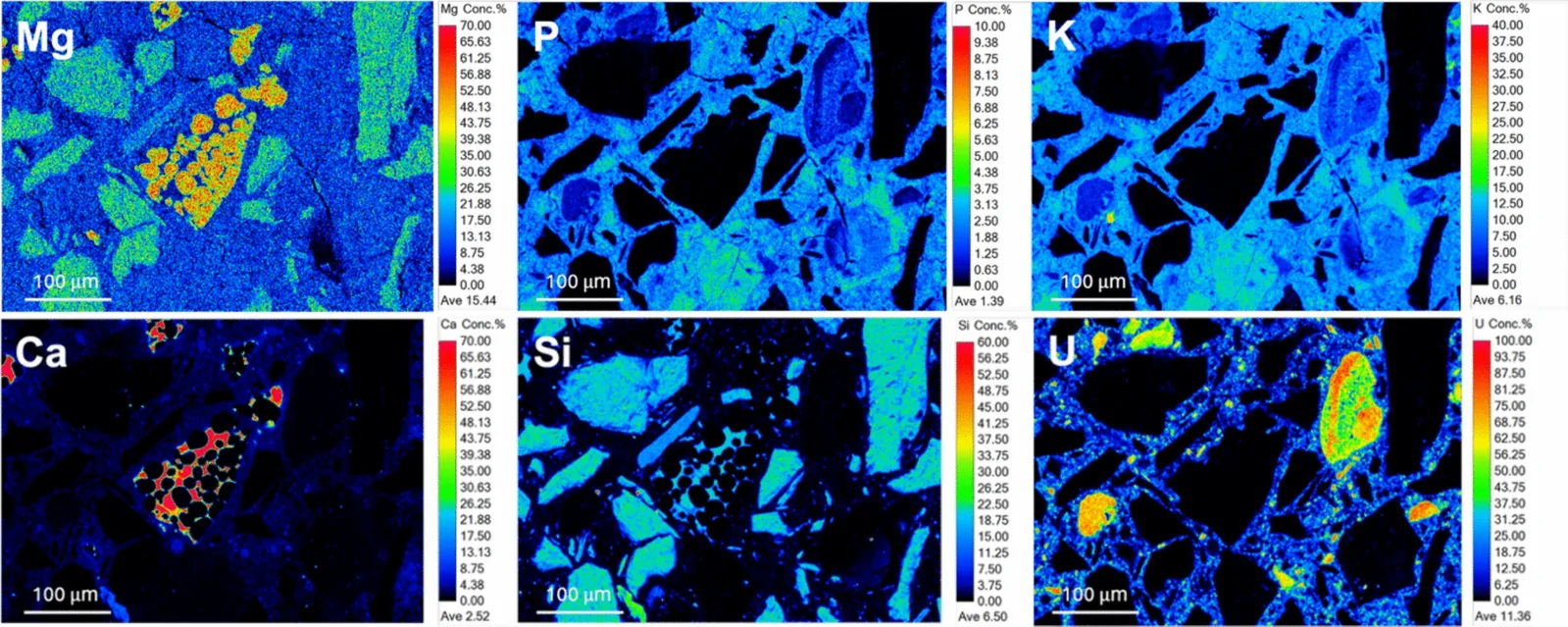
Electron probe microanalysis (EPMA) maps of magnesium potassium phosphate cement (MKPC) encapsulating U_3_O_8_ particles with data presented as corresponding wt%.

Depleted, natural and low-enriched uranium (DNLEU) waste constitutes a significant component of the UK’s low heat generating waste inventory, primarily arising from nuclear fuel cycle processes, including enrichment tails, reprocessing and miscellaneous low-enriched uranium sources. These wastes exist predominantly as UF_6_ and uranium oxides (including U_3_O_8_, UO_2_, and UO_3_), often contaminated with fission products or transuranics. They are currently stored pending a decision on reuse in the nuclear cycle or, more likely, conditioned for permanent disposal in a geological disposal facility.^27^ This section focusses on the interaction between uranium oxide-bearing waste with magnesium potassium phosphate cement (MKPC), using U_3_O_8_ as a representative legacy and process-derived wasteform. EPMA provides a valuable means to resolve the spatial distribution of uranium within the hardened cement matrix, identify reaction rims and potential secondary phases, thereby providing insight into uranium retention and redistribution over curing. Ordinary Portland Cement (OPC) is considered the standard encapsulation matrix for low and intermediate level radioactive waste due to its cost-effectiveness, adaptability to diverse waste streams and ability to maintain high pH conditions that reduce radionuclides solubility. The immobilisation of actinides, lanthanides, and certain transition-metal radionuclides in OPC matrices is attributed mainly to their chemical fixation as poorly soluble silicate hydrates and hydroxides under alkaline conditions. These reactions are complemented by adsorption or incorporation onto calcium-silicate-hydrate phases, thereby limiting radionuclide mobility.^28^ However, for the specific management of uranium oxides, MKPC may offer a superior alternative by providing a near-neutral chemical environment (typically pH 7–9), highly compatible with actinide-rich oxides, preventing the formation of soluble complexes and promoting the precipitation of stable phosphate phases. In addition, MKPC develops a dense crystalline microstructure that enhances physical encapsulation of U_3_O_8_ particles, resulting in a wasteform with high mechanical strength and low connected porosity. The MKPC was formulated based on previous studies on nuclear waste immobilization^29–31^ using high purity MgO and KH_2_PO_4_ with a molar Mg/P ratio equal to 1.7. Olivine was incorporated as a filler material (filler ratio of 0.5). Boric acid was added as a retarder (2 wt% relative to binder). The water to solid ratio was set to 0.24. 20 wt% of U_3_O_8_ was loaded into the homogenized cement paste and the sample was cured at 35°C, representing a conservative thermal environment for low heat generating waste.^32^ After curing for 90 days, a polished section was examined by EPMA (**Figure** **7**). The BSE images show the distribution of Mg, P, K, Ca, Si, and U in a selected area. Silicon mapping identifies the structural framework of angular olivine aggregates that remain chemically distinct from the surrounding cementitious matrix. The Mg and Ca distributions indicate heterogeneity in the MgO precursor, with localized enrichments suggesting the presence of partially unreacted periclase agglomerates containing residual CaO from magnesite calcination. Uranium is predominantly present as discrete U_3_O_8_ aggregates. Notably, P and K signals overlap in regions surrounding these particles, consistent with the presence of the K-struvite matrix, but also suggesting localized interaction with phosphate-rich phases. The lower concentration of U at the aggregates boundaries indicates partial diffusion of K and P into the outer regions of U_3_O_8_ particles. This interfacial zone likely enhances both, chemical bonding and mechanical adhesion between the waste particles and the cement matrix, contributing to improved immobilisation.

## Probing the long-term corrosion behavior of vitrified high level wastes

Vitrification of the UK’s high level waste (HLW) inventory is ongoing with an estimated packaged volume of 1340 m^3^.^5^ Over time, in contact with water, glass will devitrify resulting in an amorphous gel layer and assemblage of secondary minerals.^33–35^ Understanding the long-term degradation of vitrified waste is important to underpin the safety case for disposal in a GDF, including release rates of mobile radionuclides and the stability of secondary phases that might accumulate less-mobile radionuclides. Simulant HLW glasses, using nonactive surrogates for radionuclides, were produced at the laboratory and industrial scale (e.g., using lanthanides to represent actinides and radioactive lanthanide fission products). Simulants have undergone long-term dissolution tests under both laboratory and field conditions to understand the evolution of alteration layers and the distribution of radionuclide surrogates within them. EPMA has been used to provide high-resolution chemical mapping of glasses altered for up to three years in the laboratory and up to 16 years under environmentally relevant conditions.

**Figure 8:**
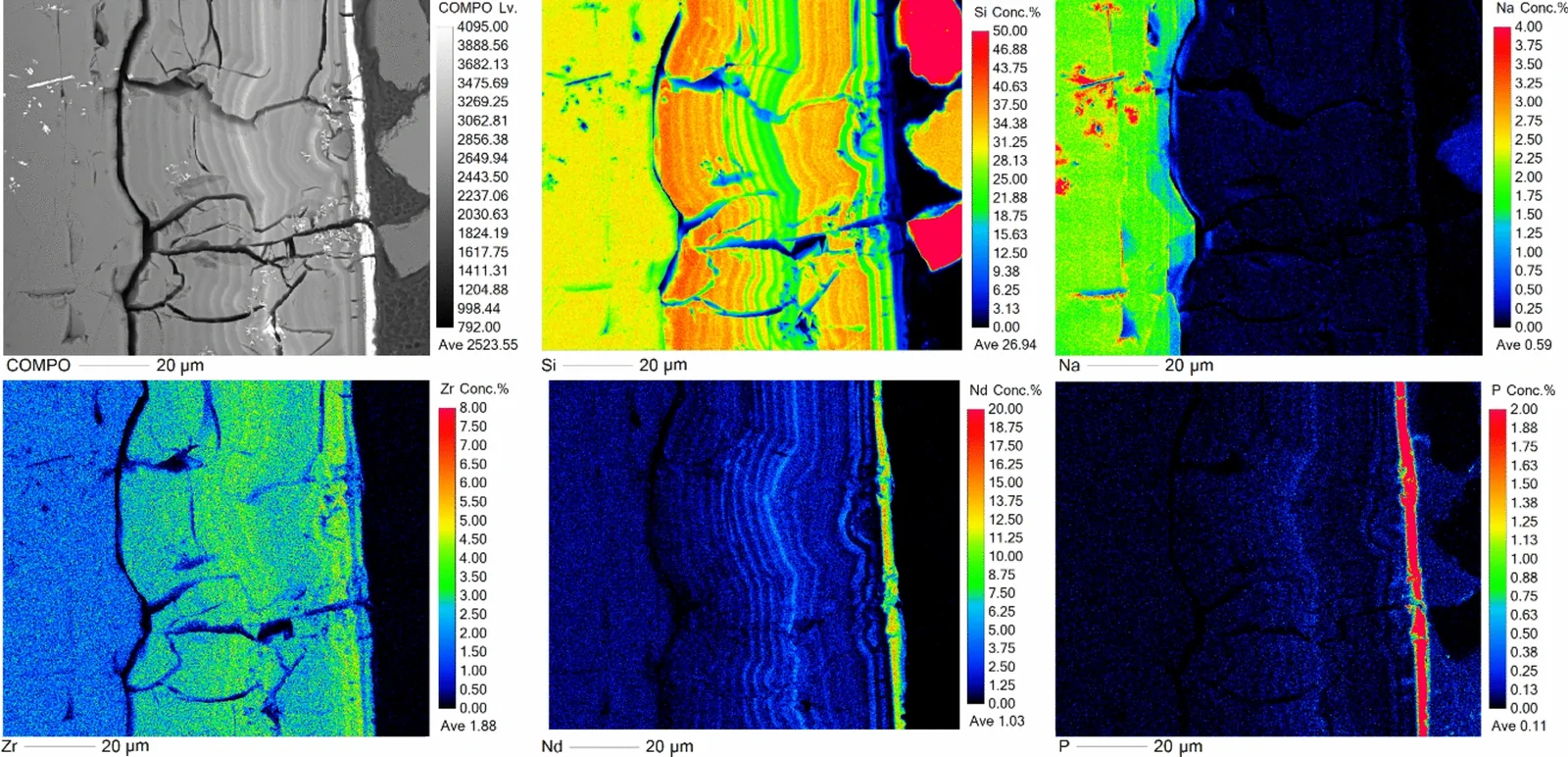
Electron probe microanalysis (EPMA) maps of simulant United Kingdom High Level Waste glass corroded in a complex natural environment with data presented as corresponding wt%.

EPMA identified chemical zoning throughout glass alteration layers where lanthanides associated with Ca and P in Ca–Ln–P-rich bands/layers (**Figure** **8**). The distribution of other elements (e.g., Fe, Al, Zr) was also observed to vary across the 40-µm alteration layer. Simultaneous mapping of the pristine glass, alteration layer and attached soil minerals provided evidence as to the contribution of elements sourced from the glass and sediment, respectively. EPMA was found to have some limitations when applied to glass samples due to the presence of light elements within the glass matrix.

**Figure 9:**
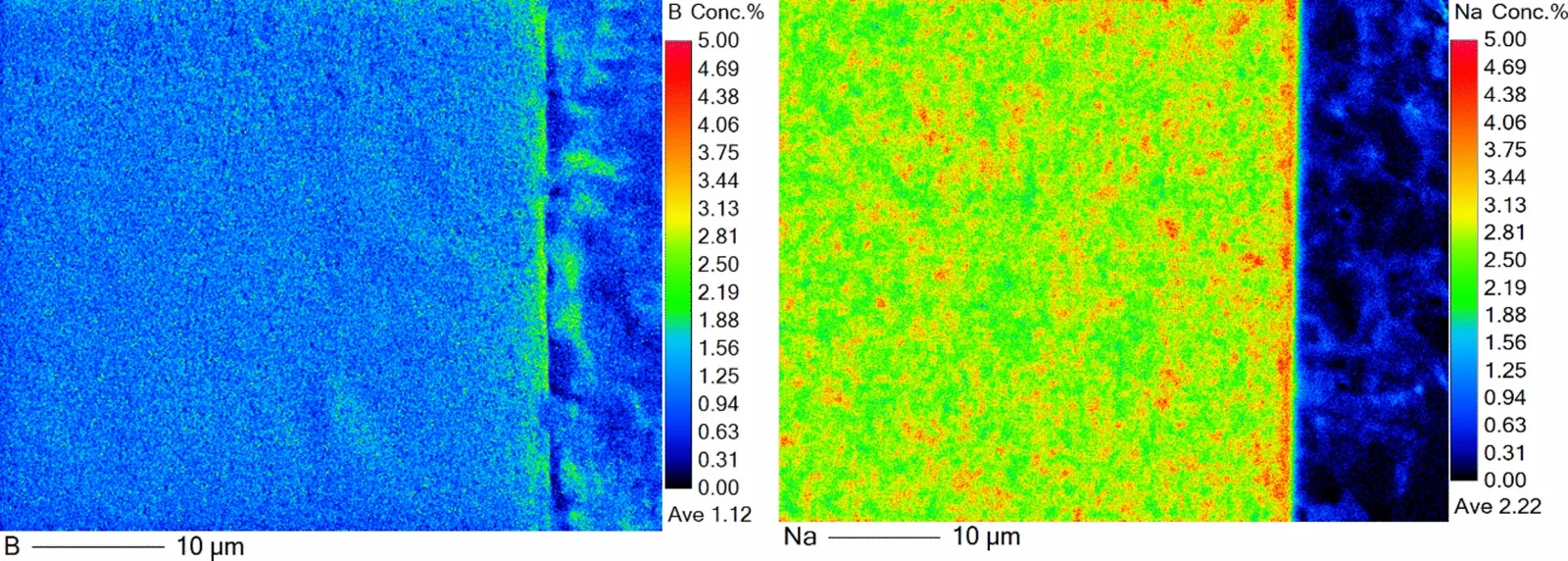
Edge effects observed when mapping mobile elements B and Na in alkali borosilicate glasses with data presented as corresponding wt%.

Light elements, including (B, alkali cations) were particularly affected by the beam dwell time and required careful calibration to achieve accurate quantitative measurements (such as comparison with other analytical techniques to ensure that underestimation was accounted for). Highly mobile elements migrate toward the sample surface under the electron beam either diffusing or volatilizing causing a rapid drop in the X-ray signal detected. Some element migration was evident, making it appear, falsely, that Na and B were enriched at the edge of the sample and sometimes around mineral inclusions (**Figure** **9**). Comparison with unaltered glass specimens helped to identify these experimental artefacts and glasses were mapped under the following conditions: accelerating voltage 15 kV, beam current 10 nA, dwell time per pixel < 1000 ms. Na was always measured first with the minimum counting time of 10 s for Na, K and Ca. There were also challenges in the simultaneous mapping of very different matrices, for example optimizing calibration standards to provide quantitative mapping of elements in the Ca–Ln–P-rich layer and the glass matrix simultaneously. Despite these challenges EPMA was an extremely useful tool in glass alteration analysis allowing the spatial distribution of elements to be determined with high resolution.

## Concluding remarks

The safe management of radioactive waste remains a scientific and engineering challenge of global significance. Immobilization using glass, ceramics, cementitious, and geopolymer wasteforms is an established practice and is often a necessary step to facilitate disposal and fabricate passively safe waste packages that are compatible with geological disposal, identified internationally as the technically mature solution to permanent waste disposal. Material characterization is fundamental to understanding wasteform composition, probing immobilization behavior, element partitioning, phase segregation and leaching performance among other properties. Nondestructive and minimally invasive techniques that provide accurate composition information and detailed phase mapping, such as EPMA, are thereby indispensable and such capabilities (especially those compatible with radiological specimens) are a vital tool to probe the chemistry of wasteform systems. Such data ultimately support the disposal of vast quantities of radioactive waste that currently exist internationally.
